# Adjunctive naturopathic care for type 2 diabetes: patient-reported and clinical outcomes after one year

**DOI:** 10.1186/1472-6882-12-44

**Published:** 2012-04-18

**Authors:** Ryan Bradley, Karen J Sherman, Sheryl Catz, Carlo Calabrese, Erica B Oberg, Luesa Jordan, Lou Grothaus, Dan Cherkin

**Affiliations:** 1 Bastyr University Research Institute (BURI), Kenmore, WA, 98028, USA; 2 Group Health Research Institute (GHRI), Seattle, WA, 98101, USA

## Abstract

**Background:**

Several small, uncontrolled studies have found improvements in self-care behaviors and reductions in clinical risk in persons with type 2 diabetes who received care from licensed naturopathic physicians. To extend these findings and determine the feasibility and promise of a randomized clinical trial, we conducted a prospective study to measure the effects of adjunctive naturopathic care (ANC) in primary care patients with inadequately controlled type 2 diabetes.

**Methods:**

Forty patients with type 2 diabetes were invited from a large integrated health care system to receive up to eight ANC visits for up to one year. Participants were required to have hemoglobin A1c (HbA1c) values between 7.5-9.5 % and at least one additional cardiovascular risk factor (i.e., hypertension, hyperlipidemia or overweight). Standardized instruments were administered by telephone to collect outcome data on self-care, self-efficacy, diabetes problem areas, perceived stress, motivation, and mood. Changes from baseline scores were calculated at 6- and 12-months after entry into the study. Six and 12-month changes in clinical risk factors (i.e., HbA1c, lipid and blood pressure) were calculated for the ANC cohort, and compared to changes in a cohort of 329 eligible, non-participating patients constructed using electronic medical records data. Between-cohort comparisons were adjusted for age, gender, baseline HbA1c, and diabetes medications. Six months was pre-specified as the primary endpoint for outcome assessment.

**Results:**

Participants made 3.9 ANC visits on average during the year, 78 % of which occurred within six months of entry into the study. At 6-months, significant improvements were found in most patient-reported measures, including glucose testing (P = 0.001), diet (P = 0.001), physical activity (P = 0.02), mood (P = 0.001), self-efficacy (P = 0.0001) and motivation to change lifestyle (P = 0.003). Improvements in glucose testing, mood, self-efficacy and motivation to change lifestyle persisted at 12-months (all P < 0.005). For clinical outcomes, mean HbA1c decreased by −0.90 % (P = 0.02) in the ANC cohort at 6-months, a −0.51 % mean difference compared to usual care (P = 0.07). Reductions at 12-months were not statistically significant (−0.34 % in the ANC cohort, P = 0.14; -0.37 % difference compared to the usual care cohort, P = 0.12).

**Conclusions:**

Improvements were noted in self-monitoring of glucose, diet, self-efficacy, motivation and mood following initiation of ANC for patients with inadequately controlled type 2 diabetes. Study participants also experienced reductions in blood glucose that exceeded those for similar patients who did not receive ANC. Randomized clinical trials will be necessary to determine if ANC was responsible for these benefits.

## Background

The use of complementary and alternative medicine (CAM) by people with type 2 diabetes exceeds that for the general population, with estimates of use as high as 72 % (excluding solitary prayer) [1-5]. Although the use of CAM in this population is high, little is known about the effectiveness of CAM practices on diabetes-related outcomes. For this reason, the Institute of Medicine has included comparative effectiveness research (CER) of CAM practices as a priority [6].

Care provided by naturopathic doctors (ND) is a particularly promising form of CAM practice for diabetes, because the ND training emphasizes assessment and understanding of medical risk, intensive dietary and lifestyle counseling, and the routine laboratory testing and medication prescribing necessary for ongoing management [7-10]. Although randomized, controlled trials are lacking, the findings from several small studies suggest elements of ND care may lead to improved cardiometabolic health in persons with type 2 diabetes. A small, uncontrolled 3-month clinical trial of ND approaches to nutrition counseling for type 2 diabetes demonstrated statistically significant reductions in hemoglobin A1c (HbA1c), and several concurrent improvements in self-care, including improved dietary adherence and improved eating behaviors [10]. Retrospective observational studies also suggest ND care reduces risk for type 2 diabetes and hypertension, including improved glucose control and reduced blood pressure, respectively [7,8]. Because recent ancillary analyses from the National Health Interview Survey (NHIS) data suggest 13 % of visits to NDs were for type 2 diabetes [11], further research is warranted to evaluate the safety and effectiveness of ND practices in diabetes.

Prior to performing a randomized clinical trial of ND approaches to care in type 2 diabetes, several research gaps had to be filled, including the collection of data on the acceptability and safety of ND practices, prospective evidence of improved outcomes, and whether the effects seemed generalizable. To fill these gaps, we conducted a one-year prospective study of ND care provided as an adjunct to usual care, i.e., adjunctive naturopathic care (ANC), delivered to primary care patients with inadequately controlled type 2 diabetes recruited from a managed care setting. We were particularly interested if the emphasis on behavioral counseling reported in past research of ND care would be evident in our study, if the receipt of behavior change recommendations would translate into improvements in patients’ behavior and/or motivation to change behavior during the observation period, and if any commensurate changes in clinical risk would be evident. In this manuscript, we report the results, including a summary of the treatment recommendations delivered by NDs, the changes in patient-reported outcome measures and standard clinical measures; and participants’ use of routine medical services during the observation year.

## Methods

### Overview of study design

We conducted a one year observational cohort study of persons with type 2 diabetes who were invited to receive up to 8 visits of adjunctive naturopathic care (ANC). Unlike an explanatory clinical trial with a defined protocol, in this pragmatic observational study we left the number and timing of visits, as well as the specific content of visits to the discretion of the ANC providers. Our outcome measures included both changes in patient-reported outcomes (PROs) and clinical laboratory risk measures, e.g., HbA1c, blood pressure, and lipids, evaluated at six month intervals during and following exposure to ND care as a whole discipline. All elements of the study were reviewed and approved by the Group Health Institutional Review Board.

### Study population

All participants were patients at Group Health (GH), a large non-profit, integrated health care system based in Seattle, WA who were not seeking ND care nor had prior experience with ND care. Our goal was to enroll 40 participants aged 21–65 years with type 2 diabetes who had the potential for meaningful improvement in clinical risk factors. We therefore recruited individuals who had a HbA1c between 7.5-9.5 % over the past year and who had at least one additional cardiovascular risk factor, i.e., those with elevated lipids (i.e., LDL > 100 mg/dl, HDL <35 mg/dl for men or <45 mg/dl for women, and/or triglycerides >150 mg/dl), high blood pressure (≥140/90 mmHg) and/or those who were overweight (BMI > 25). Because GH’s electronic health record (EHR) includes data on age, gender, clinical diagnoses, laboratory results, and dispensed medications (i.e., drug names, date of prescriptions, and refills) we were able to exclude patients who did not have lab values within our target ranges and others who were inappropriate for participation, including those who had a myocardial infarction or stroke within the past six months, a recent history of bariatric surgery, a diagnosis of severe psychiatric illness, (i.e., schizophrenia or personality disorder) and/or a diagnosis of cancer (except non-melanoma skin cancer). We also excluded patients taking insulin to reduce the variability in our small sample.

### Participant recruitment procedures for the ANC cohort

Patients meeting our inclusion criteria were mailed a letter explaining the study and a postcard to return if they had interest in participating. A research specialist contacted candidates who returned the postcard and those who remained interested and consented to participate were given instructions to complete a laboratory test to confirm their HbA1c value was between 7.5-9.5 %. Candidates confirmed to be eligible based on HbA1c testing were enrolled and administered a baseline telephone interview.

### Creation of the usual care cohort

Electronic health record (EHR) data collected from a cohort of patients who continued to obtain their usual GH care was used for comparing clinical outcome measures. Patients in this group had baseline EHR data comparable to those in the ANC group, and thus were potentially eligible to receive the ANC intervention, but they were not invited to participate. The only other known difference between the cohorts is that many of patients in the usual care cohort resided further from Seattle that those recruited to the ANC cohort.

### Recruitment of naturopathic doctors (ND), delivery of ANC, collection of data to describe treatment

We selected four naturopathic doctors (ND) licensed in WA State who had been practicing in the community for at least five years and did not have specialty practices. The NDs were instructed to deliver their typical care to participants. Study participants were given a choice of ND practice location to receive their care and provided with the necessary information to schedule their first appointment. Completing the first visit was left to each participant’s motivation and the number and timing of follow-up visits was decided between the ND and the participant. Following all of the ANC visits, a research specialist abstracted data from the chart notes using a standardized data collection form.

### Collection of patient-reported outcomes (PROs) data

PROs data were collected over telephone interviews upon enrollment and again after 6- and 12-months. Data on demographics and medical history, including year of diabetes diagnosis and history of heart failure, heart attack, stroke, and/or micro-vascular complications including neuropathy, retinopathy, cataracts, and/or nephropathy were collected at baseline. We used the Summary of Diabetes Self-Care Activities (SDSCA) instrument to measure diabetes self-care, which captures the number of days in the past week patients engaged in a variety of important self-care activities (e.g., checking glucose, regular physical activity, eating fruits and vegetables, and taking medications as recommended) [12]. Mood and depression were assessed using the PHQ-8 [13,14]. The 16-question Self-Efficacy Scale (SES) [15,16] was used to assess participant self-efficacy. Each question on the SES was scored on a 0–8 Likert scale with 0 = “Not at all confident” and 8 = “Extremely confident”. Responses to each question were combined to calculate a single composite score [17]. We measured motivation for changing self-care with the Readiness Index (RI) [18], a nine-question instrument that uses a Likert scale ranging from 1 (“Strongly disagree”) to 6 (“Strongly agree”) to assess three primary domains: evaluation of lifestyle, creating strategies for change and goal commitment. Stress from diabetes was measured using the Perceived Stress Scale (PSS), the most widely used measure of self-reported psychological stress [19]. The PSS is a state measure that asks about stress as experienced over the last month. Finally, we used the Problem Areas in Diabetes (PAID) instrument to measure diabetes-related emotional distress. The PAID score has been found, after adjustment for age, diabetes duration, and general emotional distress, to be a unique contributor to adherence to self-care behaviors [20]. In addition, higher PAID scores have been found to be associated with HbA1c independently of age, diabetes duration, general emotional distress and adherence to self-care behaviors.

We also asked participants to rate their GH diabetes care and their ND care on a 5-point scale, (1 = “Very satisfied” and 5 = “Very dissatisfied”) and their perceived effectiveness of GH and ND care *for their diabetes* on a 5-point scale (1 = “Very effective” and 5 = “Harmful”). Finally, participants were asked if their ND care changed the way they thought about their diabetes (Yes/No/Don’t know), if they had changed anything about diabetes care *as a result of* their ND care (Yes/No/Don’t know), and if they believed there was anything harmful about their ND care (Yes/No/Don’t know). We asked for an explanation for any reports of harm.

### Collection of clinical risk factor outcomes data

Laboratory tests were conducted at baseline and after 6- and 12-months for participants in the ANC cohort. Test results for HbA1c, lipids, and blood pressure were abstracted from the EHR for participants in our ANC cohort, and for all patients included in the usual care cohort. We also collected data in both cohorts on potential confounders (i.e., age, gender, baseline HbA1c, and use of sulfonylurea and/or metformin) from the EHR.

### Collection of data on use of prescription medications and utilization of medical services

EHR data were abstracted from the year prior to baseline and during the ANC observation year in order to estimate the number of new prescriptions and refills for insulin, sulfonylureas and metformin, as well as the number of primary care, nutrition and specialist visits made for members of both cohorts during the same time intervals.

### Statistical analysis

Our data analysis plan was completely developed before any analyses were conducted. All data were recorded in a master database and then analyzed using SAS statistical software version 9.2 (SAS Institute, Cary, NC). We pre-specified our primary outcome measures for both PROs and clinical outcomes*.* For PROs, we pre-specified our primary outcome measure as the mean change in the frequency of self-care activities captured by the Summary of Diabetes Self-Care Activities (SDSCA) for glucose monitoring, diet and physical activity subscales within the ANC cohort between baseline and 6-months. For clinical outcomes, we specified mean change in HbA1c within the ANC cohort as our primary measure of glycemic risk status and mean change in total cholesterol: HDL ratio within the ANC cohort as our primary measure of lipid risk status.

PRO results were analyzed as the change in mean score within the ANC cohort compared by paired, two-sided, t-tests. We used summary scores and/or composite scales for each instrument when possible. For the SDSCA, means were compared from three composite scales: glucose testing, diet and physical activity. Similarly, two composite scales were used for the RI: lifestyle and commitment. We compared means of composite scores for the SES, PAID and PSS instruments. PHQ-8 results were analyzed by calculating and comparing the change in mean score as well as the change in the proportion of patients reaching the diagnostic threshold for depression (i.e., ≥10) by chi^2^ test [13,14].

All EHR observations for HbA1c, lipids and blood pressure were combined with the results from our required laboratory testing in order to estimate the change in each measure during the time interval from 30 days prior to baseline until 14 months after baseline in the ANC cohort. For the usual care cohort, only the observations in the EHR were used to estimate changes in risk factors during the same time period. Maximum likelihood means were calculated for each measure because they account for the intra-individual correlation in repeat measures. Comparisons were made within the ANC cohort by comparing means from baseline to 6- and 12-months. Comparisons between cohorts were performed by generating random intercept linear regression models for each risk factor during the observation period, and comparing the slopes of the resulting linear estimates, adjusting for potential confounders including age, gender, baseline HbA1c, and use of sulfonylurea and/or metformin at baseline. Random intercept models were applied because they allow all of the available observations to impact the resulting linear model when the total number of observations varies between time points of interest, i.e., 6- and 12-months.

For comparisons of new prescription medications, medication refills and utilization of GH services, descriptive statistics were calculated for the year prior to baseline and for the year of ANC observation. Differences were not compared statistically as they were not specified as outcome measures, rather were calculated to provide context for any observed changes in clinical risk that may have occurred.

## Results

Forty participants (n = 40) were recruited for the ANC cohort and we identified three hundred twenty-nine patients (n = 329) as a usual care cohort for comparisons. Cohort characteristics are summarized in Table 1. Compared to our usual care cohort, the ANC cohort was slightly older, had a longer duration of GH care, was less likely to have hypertension, and more likely to take metformin. The number of HbA1c tests was also greater in the ANC cohort, though we believe this difference is an artifact of our requirement to test HbA1c before enrollment.

**Table 1:** Baseline Characteristics

Demographics and Clinical Status	ANC Cohort n = 40 % or Mean (SD)	Usual Care Cohort n = 329 % or Mean (SD)	P-value^a^
Male gender (%)	60 %	54 %	0.50
Age, years	57.6 (7.6)	54.3 (8.2)	0.02
Years of diabetes CARE at GHC	7.2 (4.2)	5.8 (4.5)	0.07
Glycemic Status			
Hemoglobin A1c, %	8.0 (0.6)	8.1 (0.8)	0.56
#A1c tests in past 12 months	2.9 (1.0)	2.2 (0.9)	0.001
Lipid Status			
Total cholesterol: HDL ratio	3.9 (1.4)	4.3 (1.3)	0.06
Total cholesterol (mg/dl)	171.6 (36.0)	181.0 (41.2)	0.18
LDL-C (mg/dl)	89.7 (29.0)	98.1 (97.2)	0.18
HDL-C (mg/dl)	47.4 (13.3)	44.1 (11.2)	0.09
Triglycerides (mg/dl)	186.8 (118.2)	205.2 (141.2)	0.43
Blood Pressure			
% ≥140/90 mmHg	22 %	33 %	0.20
% ≥130/80 mmHg	40 %	60 %	0.02
Medication Use (%)			
Any	90 %	78 %	0.08
Metformin	80 %	39 %	0.002
Sulfonylureas	65 %	72 %	0.30
Other	0 %	1 %	0.62

^a^ P-values are for chi^2^ comparisons of proportions or comparisons of maximum likelihood means by unpaired, two-sided t-tests.

On average, participants made 3.9 ± 2.1 ANC visits during the 12-month observation period. Utilization of ANC declined over the observation period by quarter with 37 (93 %), 24 (60 %), 18 (45 %) and 5 (13 %) participants using ANC during the first, second, third and fourth quarters respectively. Thirty-eight (95 %) and thirty-three (83 %) participants completed the 6- and 12-month follow-up interviews, respectively. Thirty-three (83 %) and nineteen (48 %) participants completed laboratory testing at 6- and 12-months, respectively.

Table 2 summarizes the recommendations made to participants by the ND providers according to their chart notes. Recommendations to change self-management practices were very common (95 % of patients received some advice), and usually included reinforcement of advice to monitor glucose (64 %) and to adhere to prescription medications (74 %). Recommendations for dietary changes, including changes to diet composition (95 %) and behavior related to eating (92 %), were also very common. Most participants were advised to increase physical activity (100 %) and given specific stress management recommendations (59 %). Although there was considerable overlap in the recommendations for general categories of self-care, and some specific recommendations (e.g., increase dietary protein (54 %), increase vegetables (46 %) and add walking (79 %)), many recommendations were given to small proportions of participants, suggesting individualization. Finally, recommendations for dietary supplements were common (74 %), with omega-3 fatty acids from fish being the most common (56 %) followed by chromium (46 %), multi-vitamin (44 %), fiber (36 %), and vitamin D3 (26 %).

**Table 2:** Summary of ANC Treatment Recommendations

**Category of Treatment Recommendations**	**Patients receiving advice** **during at least one visit-** **n (%) of 39 total patients**	**Frequency of advice** **given to each patient-** **Mean % of visits including advice**
		
Any	37 (95 %)	89 %
Reinforce medication adherence	29 (74 %)	63 %
Self-monitoring of glucose	25 (64 %)	53 %
		
Any	37 (95 %)	91 %
Mindful eating behavior	36 (92 %)	88 %
Increase protein	21 (54 %)	45 %
Increase vegetables	18 (46 %)	31 %
Increase fiber	17 (44 %)	28 %
Reduce dietary cholesterol/fat	11 (28 %)	16 %
Reduce sugar	10 (26 %)	14 %
Lower glycemic index	9 (23 %)	15 %
Increase PUFA	9 (23 %)	12.5 %
Lower glycemic load	7 (18 %)	10 %
Increase legumes	7 (18 %)	9 %
Increase fruit	6 (15 %)	9 %
Reduce trans fats	6 (15 %)	9 %
Increase herbs/spices	6 (15 %)	11 %
Increase soy	5 (13 %)	8 %
Increase tea	4 (10 %)	7 %
		
Any	39 (100 %)	93 %
Walking	31 (79 %)	66 %
Aerobic	17 (44 %)	28 %
Resistance	10 (26 %)	15 %
		
Any	23 (59 %)	42 %
Deep-breathing exercises	8 (21 %)	11 %
Meditation	6 (15 %)	9 %
Yoga	4 (10 %)	7 %
Other	5 (13 %)	5 %
		
Any	29 (74 %)	60 %
Omega-3 fatty acids	22 (56 %)	41 %
Chromium	18 (46 %)	26 %
Multivitamin with B-complex	17 (44 %)	23 %
Fiber	14 (36 %)	20 %
Vitamin D	10 (26 %)	13 %
*Cinnamomum cassia* (cinnamon)	7 (18 %)	12 %
Vitamins C and E	7 (18 %)	11 %
Probiotics	6 (15 %)	9 %
Bioflavonoid/polyphenol	6 (15 %)	8 %
*Gymnema sylvestre*	5 (13 %)	9 %
Coenzyme Q10	4 (10 %)	7 %
Other	<4 (<10 %)	<5 %

### Changes in patient-reported outcome measures

**Figure 1:**
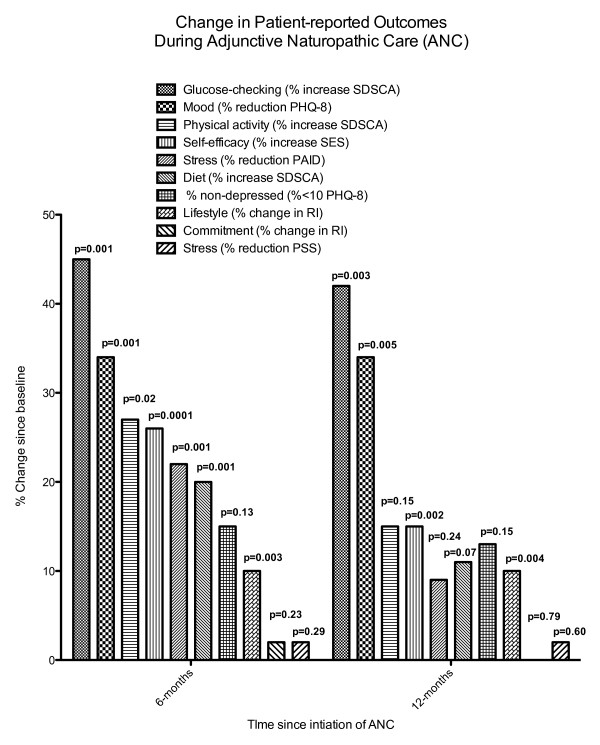
**Changes in patient-reported outcomes during ANC. **Results are reported as % change since baseline for each instrument according to the legend. P values report the results of comparisons of mean scores by two-sided, paired t-tests. SDSCA = Summary of Diabetes Self-Care Activities; PHQ-8 = 8-question Personal Health Questionnaire; SES = Self-Efficacy Scale, PAID = Problem Areas in Diabetes; RI = Readiness Index; PSS = Perceived Stress Scale.

At 6-months, significant changes were found in most patient-reported outcomes (Figure 1), including increased frequency of self-care practices (i.e., glucose testing, following a healthy diet and physical activity), increased self-efficacy, reduced diabetes problem areas, improved motivation for changing lifestyle and improved mood. Little change was found in the commitment subscale of the Readiness Index, or in the composite score of the Perceived Stress Scale (P > 0.05 for both). Despite relatively little utilization of ANC after the 6-month interview, several significant changes persisted at the 12-month interview, including increased glucose testing, improved mood, increased self-efficacy, and increased motivation for changing lifestyle.

### Changes in clinical risk factors

**Figure 2:**
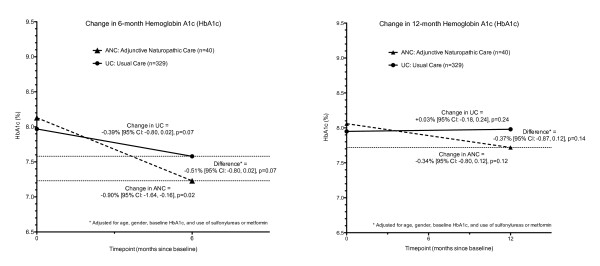
**Changes in hemoglobin A1c during ANC compared to usual care.** P values for within cohort change were computed using maximum likelihood estimates of mean HbA1c (random intercept, mixed effects model) and correspond to within cohort 2-sided t-tests; between cohort comparisons of mean change from baseline are from the random slope and intercept mixed models (maximum likelihood, similar to unpaired t-test of mean change over the observation period), adjusted for age, gender, baseline HbA1c and use of sulfonylureas and/or metformin at baseline. ANC = Adjunctive Naturopathic Care, UC = Usual Care.

HbA1c was significantly lower in the ANC cohort 6-months after baseline (−0.90 %, 95 % CI: -1.64 %, -0.16 %; p = 0.02) and reductions in HbA1c favored the ANC cohort compared to the usual care cohort after adjustments for age, gender, baseline HbA1c and use of sulfonylureas and/or metformin (Figure 2). Improvements in glycemic control were still greater at 12-months in the ANC cohort, but the differences were smaller and not statistically significant. Lipid-associated risk changed minimally in both cohorts during the observation period (change in total cholesterol: HDL ratio = −0.23 vs. -0.15, P = 0.49 for ANC versus usual care, adjusted for age, gender, baseline HbA1c and use of sulfonylurea and/or metformin). Changes in other lipid and blood pressure measures were minimal between cohorts and differences did not reach statistical significance at either 6- or 12-months (P > 0.05 for within and between cohort changes in total cholesterol, LDL-C, HDL-C, triglycerides, systolic and diastolic blood pressure).

### Changes in prescription medications and utilization of medical services

Use of oral medications and insulin increased in the ANC cohort during the 12-month observation period, with three patients starting on sulfonylureas, three patients starting on metformin and six patients starting on basal insulin. The total number of prescription refills also increased in the ANC cohort on average during the observation period from 4.7 ± 2.2 per year during the year prior to baseline compared to 5.9 ± 2.7 per year during the ANC observation period, whereas refills did not change in the usual care cohort (i.e., 4.2 ± 3.0 refills/year during the prior year compared to 4.0 ± 3.2 refills during the ANC observation period).

Utilization of GH primary care services increased in the ANC cohort from 5.0 ± 4.5 visits/year during the year prior to baseline to 6.5 ± 5.6 visits/year during the observation period. In contrast, use of primary care by members of the usual care cohort remained unchanged, i.e., 5.5 ± 4.2 during the year prior to baseline vs. 5.5 ± 5.4 visits/year during the observation period. Utilization of nutrition, emergency room and specialty care services did not change appreciably in either cohort.

### Satisfaction, perceived benefits and effectiveness of care

Participants reported nearly equivalent satisfaction with their ANC as with their usual GH care for diabetes at the 12-month interview (mean satisfaction = 2.0 ± 1.1 (ANC) and 1.8 ± 0.8 (GH)), which was virtually unchanged from their baseline report (mean satisfaction = 2.0 ± 0.8). Participants reported their GH care was effective for their diabetes (mean rating 1.9 ± 0.8), with ratings of the effectiveness of ANC slightly higher, suggesting less perceived effectiveness (2.6 ± 1.0). However, the majority of participants perceived benefit from their ANC. At the12-month interview, 63 % of participants reported they changed the way they think about their diabetes as a result of their ANC and 72 % of participants stated that they had changed their diabetes care as a result of their participation.

### Adverse effects of ANC

At 6-months, four participants attributed harm to some element of their ANC, including two comments regarding the high costs of dietary supplements, one comment on an apparent increase in blood pressure, and one comment regarding a possible adverse reaction to a dietary supplement. At 12-months, only two participants attributed harm to some element of their ANC, including a possible adverse reaction to iron supplementation and one participant noting feelings of guilt for not following recommendations.

## Discussion

The results reported here provide the first prospectively collected estimates of change in outcomes during and following the delivery of ND care to people with type 2 diabetes. Provision of ANC was associated with a variety of improvements in diabetes self-management including: increased self-monitoring of blood glucose, improved diet, increased physical activity, greater self-efficacy, improved mood and reduced problem areas in diabetes. Glucose control also improved in the ANC cohort, while remaining unchanged in the usual care cohort. Although we attempted to minimize confounding between groups by performing adjusted analyses for our primary clinical comparisons, our study was observational and therefore these encouraging findings cannot be causally attributed to ANC. Nevertheless, several factors suggest probable causation. Foremost, causation is supported by the temporality of the observations with the greater improvements corresponding to the period of greater utilization of ANC, i.e., the first 6-months. Secondly, the treatment recommendations delivered by the NDs closely correspond to the types of changes reported by participants. Finally, the majority of participants reported they made self-care changes *as a result of* their ANC.

Although there may have been overlap in the content of ANC visits with that of visits to primary care providers, diabetes educators, and nutritionists during the typical GH care for diabetes, any benefits from the delivery of these services to participants during the study period would have been included in our estimates of change in both groups.. However, even if ANC was responsible for the improvements measured in this study, the simultaneous increases in utilization of usual care medical services in the ANC group make it impossible to distinguish between direct effects of the ANC intervention and indirect effects of ANC, which may have stimulated patient re-engagement in their health and health care more generally.

Permanently changing behaviors is notoriously difficult for patients. Comparable to several large clinical trials that have tested behavior-targeted interventions, our results suggest the greatest changes in behavior and clinical risk occurred during the most intensive phase of the intervention (months 1 through 6) and then decreased thereafter [21-24]. Yet, unlike most previous trials, our intervention was delivered within the context of routine care by physician-level providers and not according to a standardized protocol. This lack of treatment standardization or inclusion of special incentives for patients to adhere to a fixed visit schedule likely resulted in lower utilization, and possibly fewer benefits, than may have occurred with a more standardized intervention. Typical barriers for patients to adopt longer-lasting behaviors include depression, reduced self-efficacy and low patient motivation [15,25,26]. Therefore, the persistence in improvements in mood, self-efficacy and motivation to change lifestyle beyond the period of greatest utilization of ANC, , is a promising sign that longer lasting changes in patient behaviors may be possible through optimization of ND visit content and frequency.

The pragmatic delivery of care, combined with collection of patient-reported and clinical outcomes following a CAM intervention, are unique features and considerable strengths of the study design because they provide a multi-faceted view of the potential real world effects of ANC implemented within usual care in a real world clinical setting (vs. the very structured, but often untranslatable, components of a clinical trial protocol). Inviting patients from a managed care setting is also a unique feature of this study compared to past research on ND care in diabetes because the patients were not explicitly seeking additional care, and therefore our results may be more generalizable than past results from samples of self-selecting patients [7]. However, these differences also make it difficult to compare the changes in HbA1c we observed in this study to those reported by earlier evaluations of ND care. Prior retrospective assessments of ND care reported a mean change of −0.65 % in patients at an academic clinic [7]. However, patients may have been more motivated to change self-care than the participants in the current study because they self-selected for ND care. Also, on average, the patients included in that study completed eleven visits over a twenty-seven month observation period compared to only four visits over a twelve-month period in the current study. A recent uncontrolled trial of a ND nutritional program also found a reduction in HbA1c (−0.4 %) after just 3 months [10], but this study included a well-developed protocol in contrast to the pragmatic, real-world approach we used in the current study. The generally positive findings of all of the observational studies of ND care support the need for carefully designed randomized trials.

One important limitation in the generalizability of our findings is the ND care in this study was applied as an adjunct to usual care, which may have limited its benefits. It remains unknown if the changes in PROs or clinical risk could have been increased or extended had the co-utilization of usual care and ND care been formally coordinated (or “integrated”) or if ND care had been offered as a primary care option. Because the delivery of behavioral change counseling is infrequent in typical primary care [27,28], very limited data are available regarding the potential impact of an ongoing, routine emphasis on behavioral change coupled to routine clinical services. Future research should investigate the formal integration of usual and ND care, and extend the treatment duration of ND care, in order to further evaluate its potential for promoting lasting behavior change.

There are several additional limitations to our study: 1) because our study population was mainly Caucasian and relatively well educated, the results may not apply to regions with different demographic characteristics, 2) our study was conducted in a managed care setting that imposed some constraints on ND practices (e.g., NDs could not directly order additional laboratory tests for GH patients or prescribe medications without approval of the patient’s GH primary care provider), and 3) because dietary supplements are not covered by insurance, many participants reported they did not use the supplements recommended by the ND, which may have reduced the potential clinical benefits of ANC. For example, although the clinical effectiveness of many dietary supplements remains unknown, omega-3 fatty acids have strong evidence for improving outcomes in people with high cardiovascular risk and small, randomized trials suggest chromium, cinnamon and coenzyme Q10 reduce blood glucose and improve other risk factors for developing complications of diabetes [29-33].

Based on our observations of improved glucose control, self-care, self-efficacy, and mood plus reduced problem areas in diabetes after initiating ANC, future research on ANC is justified and should employ a randomized, controlled trial design that permits determination of causality. To fully evaluate the potential value of ANC, randomized comparative effectiveness trials should compare unrestrained, “whole-system” ANC including dietary supplements and a full scope of practice with usual care. Additional pragmatic trials should compare “best practice” ND protocols to unconstrained ND care as it is practiced in the community. Finally, because of the high frequency of recommendations for dietary supplements in ND practice, and emerging evidence of potent clinical effects from placebos [34], careful consideration is needed for how to best evaluate the effectiveness of the highly variable dietary supplement recommendations made by NDs in practice.

Despite the need for continued research on ND approaches, we believe the results of this study have important implications for patients’ diabetes care, especially for patients interested in trying ANC. Our findings suggest numerous possible benefits and minimal risk for patients willing to use ANC. Our findings also suggest that ANC does not disrupt patients’ engagement in usual care and, in fact, may increase it. Finally, the observed increases in use of primary care services and medications for diabetes indicate that ANC was used as a *complement* to usual care and not as an *alternative*. These findings should be reassuring to usual care providers concerned that ANC may negatively impact medical care for type 2 diabetes—our findings suggest just the opposite.

## Conclusions

Significant improvements in key diabetes health indicators, including patient reported and clinical outcomes, were found during a one-year period of adjunctive naturopathic care (ANC) in a cohort of patients with inadequately controlled type 2 diabetes. It remains unclear if the observed improvements in self-care were responsible for the observed reductions in clinical risk. Randomized clinical trials are required to determine if the observed changes were caused by exposure to ANC and to evaluate the effects of ANC unconstrained by setting, cost and scope of practice.

## Competing interests

The authors have no competing interests to report.

## Author’s contributions

Authors contributed to this manuscript in the following ways: RB, DC and KS wrote the manuscript; LG and JL evaluated the data and wrote the statistical analysis section; and RB, DC, KS, SC, EO, and CC edited and reviewed the manuscript. All authors read and approved the final manuscript.

## Pre-publication history

The pre-publication history for this paper can be accessed here:

http://www.biomedcentral.com/1472-6882/12/44/prepub
